# Influence of aberrant right hepatic artery on survival after pancreatic resection for ductal adenocarcinoma of the pancreatic head

**DOI:** 10.1007/s00423-024-03296-x

**Published:** 2024-04-08

**Authors:** Ekaterina Petrova, Elena Mazzella, Katrin Eichler, Tatjana Gruber-Rouh, Falko Schulze, Wolf O. Bechstein, Andreas A. Schnitzbauer

**Affiliations:** 1 Department of General, Visceral, Transplant, and Thoracic Surgery, University Hospital, Goethe University Frankfurt, Theodor-Stern-Kai 7, Frankfurt, 60596 Germany; 2 Institute for Diagnostic and Interventional Radiology, University Hospital, Goethe University Frankfurt, Theodor-Stern-Kai 7, 60596 Frankfurt am Main, Germany; 3 Dr. Senckenberg Institute of Pathology, University Hospital, Goethe University Frankfurt, Theodor-Stern-Kai 7, 60596 Frankfurt, Germany

**Keywords:** Pancreas, Pancreatic cancer, Hepatic artery, Pancreatic surgery

## Abstract

**Purpose:**

The presence of an aberrant right hepatic artery (aRHA), arising from the superior mesenteric artery, is a common variant of the liver vascular anatomy. Considering that tumor spread occurs along vessels, the question arises, whether the presence of an aRHA influences the oncologic outcome after resection for cancer of the pancreatic head.

**Methods:**

Patients with ductal adenocarcinoma of the pancreatic head, who underwent resection from 2011 to 2020 at the Frankfurt University Hospital, Germany, were analyzed retrospectively. Surgical records and computed tomography imaging were reviewed for the presence of aRHA. Overall and disease-free survival as well as hepatic recurrence were analyzed according to the presence of aRHA.

**Results:**

aRHA was detected in 21 out of 145 patients (14.5%). The median overall survival was 26 months (95%CI 20.8–34.4), median disease-free survival was 12.1 months (95%CI 8.1–17.3). There was no significant difference in overall survival (26.1 versus 21.4 months, adjusted hazard ratio 1.31, 95%CI 0.7–2.46, *p* = 0.401) or disease-free survival (14.5 months versus 12 months, adjusted hazard ratio 0.98, 95%CI 0.57–1.71, *p* = 0.957) without and with aRHA. The hepatic recurrence rate was 24.4.% with conventional anatomy versus 30.8% with aRHA (adjusted odds ratio 1.36, 95%CI 0.3–5.38, *p* = 0.669). In the multivariable analysis, only lymphatic vessel invasion was an independent prognostic factor for hepatic recurrence.

**Conclusions:**

The presence of an aRHA does not seem to influence the long-term survival and hepatic recurrence after resection for ductal adenocarcinoma of the pancreatic head.

## Introduction

The arterial vessels of the liver are in anatomical proximity to the pancreatic head. Tumor infiltration, accidental injury during resection, or postoperative bleeding from the liver arteries could impact oncologic and surgical outcome after resection for pancreatic ductal adenocarcinoma (PDAC). In addition to the most frequent variant of the hepatic arterial anatomy with a single common hepatic artery arising from the celiac trunk, there are other variations such as replaced or accessory arteries, with origin from the superior mesenteric artery, the left gastric artery, the gastroduodenal artery, or the aorta.[1–3] A systematic classification of the anatomic variants of the hepatic arterial supply has been published by Hiatt et al. in a study of 1000 cases of liver harvesting for transplantation.[4] The second most common variant described by Hiatt was the presence of an accessory or a replaced right hepatic artery, arising from the superior mesenteric artery (Hiatt Type 3) which was encountered in 10.6 % of cases. With an accessory right hepatic artery there is a dual arterial supply of the right liver lobe from the celiac trunk and the superior mesenteric artery, whereas a replaced right hepatic artery arising from the superior mesenteric artery is the only arterial vessel suppling the right liver lobe.

The impact of such an aberrant right hepatic artery (aRHA) on the long-term overall survival (OS) or disease-free survival (DFS) after resection for PDAC of the pancreatic head is a matter of controversy. Eshuis et al. showed in a cohort of 790 patients no significant difference in long-term survival in patients undergoing pancreatoduodenectomy for PDAC, regardless of the presence of an aRHA.[5] Similarly, other retrospective studies showed no difference in survival outcomes.[6–8] On the contrary, Mangieri et al. demonstrated an association between the presence of an aRHA and hepatic recurrence as well as shorter DFS and OS.[9] 

The aim of the present study is to analyze the impact of the presence of an aRHA on OS, DFS, and hepatic recurrence as well as the association with common clinicopathological parameters in patents that undergo resection for PDAC of the pancreatic head.

## Materials and methods

### Study design

This is a retrospective, explorative, single-center study. It was approved by the ethics committee of the Faculty of Medicine, Goethe-University Frankfurt, Germany (Reference number 2023-1207).

### Patients

Patients, who underwent pancreatic head resection or total pancreatectomy for PDAC of the pancreatic head at the University Hospital Frankfurt, Goethe University Frankfurt, Germany in the years 2011 to 2020, were selected. Data was retrieved from the patients` records. The presence of distant metastases at the time of resection was a predefined exclusion criterion. Patients with neoadjuvant treatment were subsequently excluded, due to the small number (eight, of whom only one with aRHA).

### Clinicopathological parameters

Operation reports and tomography imaging (computed or magnetic resonance tomography) were reviewed for the presence of an aRHA. Pathology reports were reviewed for the total number of lymph nodes retrieved, number of positive lymph nodes, and tumor size. Lymph node ratio, defined as the ratio of the number of positive lymph nodes to the total number of lymph nodes retrieved, was calculated. The TNM classification was updated according to the Union for International Cancer Control (UICC), 8^th^ edition.[10] Resection margin status was defined as R0 (no tumor cells) versus R1 (tumor cells) at the resection margin on microscopic examination.

Associations between the presence of an aRHA and the parameters sex, age at the time of resection, T-stage, grade, N-stage, lymph node ratio, lymphatic vessel invasion, blood vessel invasion, neural invasion, and resection margin status were explored.

### Survival

OS was defined as time in months from the day of operation to the day of death. Patients alive at the date of last follow-up were censored. DFS was defined as time in months from the day of surgery until the day of tumor recurrence or death. Patients alive and tumor-free at the date of last follow-up were censored. Univariable survival analysis for OS and DFS according to the presence of aRHA was performed. Multivariable survival analysis with adjustment for T-stage, N-stage, grade, resection margin status, lymphatic vessel invasion, blood vessel invasion, and aRHA was performed for OS and DFS. Patients with in-hospital mortality were excluded from the survival analysis.

### Recurrence

Most of the patients were followed-up about every 6 months with physical examination, laboratory tests and some modality of cross-sectional imaging. Evidence of recurrence was either radiological or pathological. Patterns of recurrence were classified according to site as liver, lung, local, other site, or multiple sites. Hepatic recurrence was defined as the manifestation of liver metastases as the first site of tumor recurrence. Patients with follow-up of less than 24 months, who did not reach the endpoint (death or tumor recurrence) or with unknown recurrence status, were excluded. Univariable and multivariable analysis of the association of aRHA with hepatic recurrence was performed.

### Statistical analysis

Statistical analysis was performed with R version 3.6.3.[11] Descriptive statistics with absolute number and percentage of total for categorical variables and median and range for continuous variables was performed. Logistic regression was used in the univariable analysis for association of aRHA and other parameters. Median follow-up was estimated using the reversed Kaplan-Meier method. Univariable survival analysis was performed with the Kaplan-Meier method and the log-rank test. Multivariable survival analysis was performed using Cox regression. Logistic regression was applied for the analysis of the association between aRHA and hepatic recurrence. Significance level of 0.05 was assumed. Confidence intervals (CI) of 95% are given.

## Results

Out of 468 pancreatic resections, 145 upfront resections for PDAC of the pancreatic head were identified. Of those, 123 (84.8%) were pancreatoduodenectomies and 22 (15.2%) total pancreatectomies. Median age at operation time was 70.4 years (range 40.3 - 87.1) and 46.2% of the patients were female. None of the patients had distant metastases at the time of resection. In 21 (14.5%) patients aRHA was present.[Table 1] Of those, 11 had a replaced right hepatic artery, whereas 10 had an accessory hepatic artery from the superior mesenteric artery. In none of the patients aRHA resection due to oncological considerations was performed. In one case, where the tumor was abutting the vessel, divestment of the aRHA was performed. There was one case with an accidental injury and a consequent reconstruction of the aRHA intraoperatively.

**Table 1:** Descriptive statistics of clinical and pathological parameters

Parameter	N (%)/median (range)
age [years]	70.4 (40.3-87.1)
sex	
female	67 (46.2)
male	78 (53.8)
operation type	
PD	123 (84.8)
TPE	22 (15.2)
aRHA	21 (14.5)
pT	
T1	22 (15.2)
T2	88 (60.7)
T3	33 (22.8)
T4	2 (1.4)
pN	
N0	44 (30.3)
N1	57 (39.3)
N2	44 (30.3)
LNR	0.1 (0 -3.2)
neural invasion	122 (84.1)
blood vessel invasion	21 (14.5)
lymphatic vessel invasion	87 (60)
grade	
G1	2 (1.4)
G2	97 (66.9)
G3	46 (31.7)
resection margin	
R0	117 (80.7)
R1	28 (19.3)
adjuvant therapy	78 (53.8)
morbidity CDC≥3	65 (44.8)
total	145 (100)

aRHA – aberrant right hepatic artery, CDC – Clavien-Dindo-classification, LNR – lymph node ratio, PD – pancreatoduodenectomy, TPE – total pancreatectomy

The univariable analysis showed no association between the presence of an aRHA and other common clinical and histopathologic parameters [Table 2].

**Table 2:** Univariable logistic regression analysis of association of the presence of an aRHA with other clinical and oncological parameters

Parameter	N (%)		OR (95% CI)	p - value
	non-aRHA	aRHA		
age				
≤70	65 (52.4)	7 (33.3)		
>70	59 (47.6)	14 (66.7)	2.2 (0.86 - 6.16)	0.112
sex				
female	60 (48.4)	7 (33.3)		
male	64 (51.6)	14 (68.2)	1.87 (0.73 - 5.24)	0.206
pT				
T1	17 (13.7)	5 (23.8)		
T2	78 (62.9)	10 (47.6)	0.44 (0.14 - 1.55)	0.173
T3/T4	29 (23.4)	6 (28.6)	0.7 (0.18 - 2.77)	0.604
pN				
N0	37 (29.8)	7 (33.3)		
N1	47 (37.9)	10 (47.6)	1.12 (0.39 - 3.36)	0.828
N2	40 (32.3)	4 (19)	0.53 (0.13 - 1.9)	0.339
LNR >0.2	33 (26.6)	5 (23.8)	0.86 (0.27 – 2.4)	0.787
grade				
G1/G2	85 (68.5)	14 (66.7)		
G3	39 (31.5)	7 (33.3)	1.09 (0.39 - 2.84)	0.864
neural invasion	104 (83.9)	18 (85.7)	1.15 (0.35 - 5.25)	0.831
blood vessel invasion	19 (15.3)	2 (9.5)	0.58 (0.09 - 2.24)	0.49
lymphatic vessel invasion	75 (60.5)	12 (57.1)	0.87 (0.34 - 2.28)	0.773
resection margin				
R0	100 (80.6)	17 (81)		
R1	24 (19.4)	4 (19)	0.98 (0.26 – 2.94)	0.974
adjuvant therapy	69 (55.6)	9 (42.9)	0.6 (0.23 to 1.51)	0.28
morbidity CDC≥3	55 (44.4)	10 (47.6)	1.14 (0.44 - 2.9)	0.781
aRHA – aberrant right hepatic artery, CDC – Clavien-Dindo-classification, CI – confidence interval, LNR – lymph node ratio, OR – odds ratio				

### Survival

Overall, 133 patients were included in the survival analysis. Estimated median follow-up was 61.7 (95%CI 38.9 – 67.3) months. Forty-three (32.3%) patients were alive on last follow-up.

**Fig. 1:**
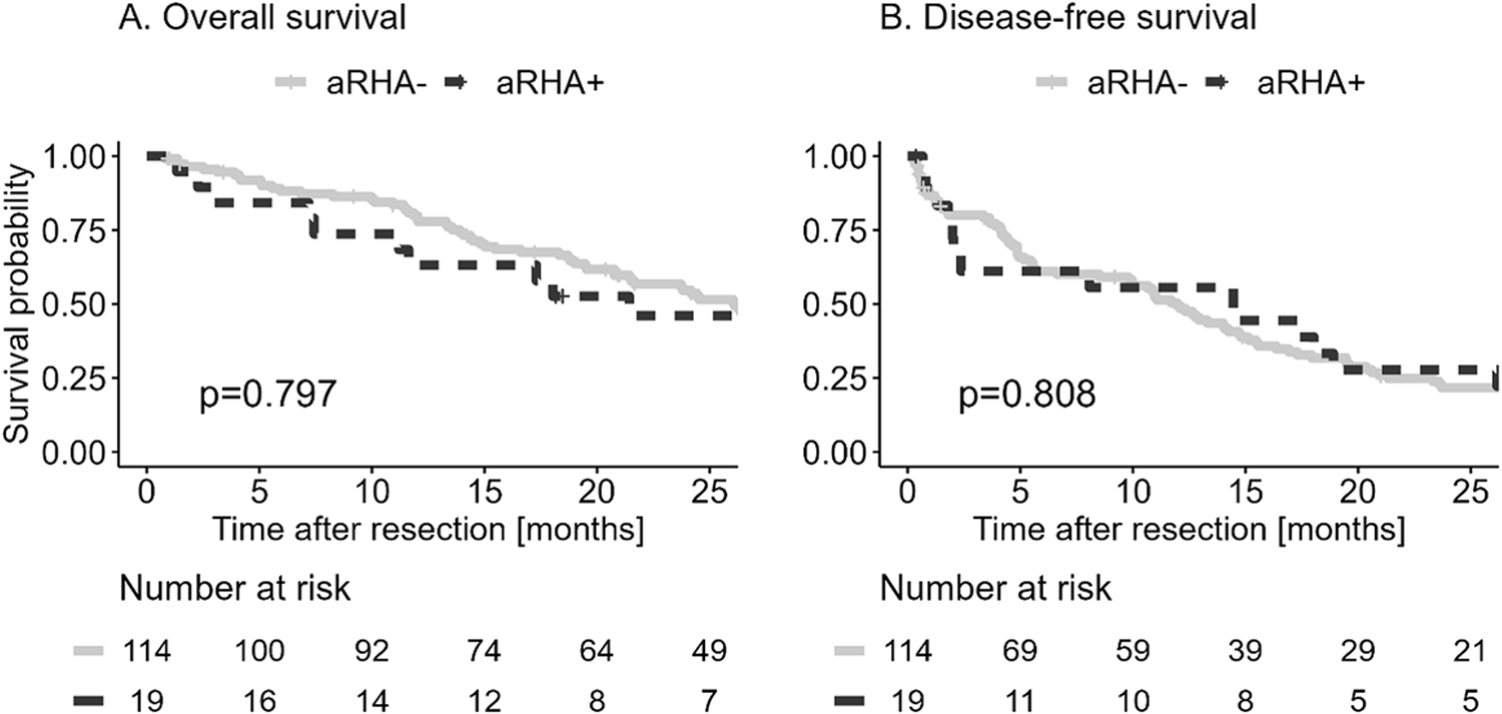
Kaplan-Meier curves of overall and disease-free survival according to the presence of an aberrant right hepatic artery (aRHA)

The estimated median OS was 26 months (95%CI 20.8 - 34.4), estimated median DFS was 12 months (95%CI 8.5 -14.5). Patients with an aRHA had shorter estimated median OS with 21.4 months (95%CI 11.2 - 49.6) compared to patients without aRHA with 26.1 months (95% CI 21.4 - 36.3), but the difference was not statistically significant with hazard ratio (HR) 1.1, 95% CI 0.6 - 1.9, p = 0.797. [Fig. 1A] In the multivariable OS survival analysis, positive resection margin R1, T stage and adjuvant therapy had prognostic significance. [Table 3] Adjuvant therapy and T-stage were prognostic factors for DFS in the multivariable analysis. [Table 3] Again, the presence of aRHA was not associated with DFS neither in the univariable analysis (HR 0.9, 95% CI 0.6 - 1.6, p = 0.808) [Fig. 1B], nor in the multivariable analysis (adjusted HR 0.95, 95% CI 0.57 - 1.71, p = 0.957) [Table 3].

**Table 3:** Multivariable survival analysis. N=133

	OS			DFS		
Parameter	Estimated median OS in months (95%CI)	HR (95%CI)	*p* - value	Estimated median DFS in months (95%CI)	HR (95%CI)	*p* - value
aRHA						
not present	26.1 (21.4 - 36.3)			12 (8.5 - 14.7)		
present	21.4 (11.2 - 49.6)	1.31 (0.7 - 2.46)	0.401	14.5 (2 - 18.9)	0.98 (0.57-1.71)	0.957
pT						
T1	36.3 (24.4 - NA)			20.6 (18 - 41)		
T2	28.5 (21.4 - 37.9)	2.61 (1.26 - 5.43)	0.01	12.1 (8.1 - 14.7)	2.78 (1.55-5)	0.001
T3/T4	18.7 (13.6 - 21.5)	2.92 (1.3 - 6.58)	0.01	4.5 (1.8 - 10)	3.26 (1.62-6.54)	0.001
pN						
N0	39.7 (23.8 - 97.6)			19.6 (12.8 - 26.9)		
N1	26.1 (14.5 - 34.4)	1.62 (0.85 - 3.07)	0.139	12.1 (4.9 - 14.5)	1.33 (0.77-2.32)	0.31
N2	19.5 (16.6 - 30)	1.74 (0.81 - 3.76)	0.157	5.5 (3.9 - 12)	1.43 (0.77-2.66)	0.251
grade						
G1/G2	26.1 (21.4 - 36.3)			12.8 (10 - 16.4)		
G3	21.7 (16.6 - 37.9)	1.17 (0.73 - 1.86)	0.512	10.7 (4.9 - 12.9)	0.96 (0.63-1.48)	0.857
lymphatic vessel invasion						
L0	28.5 (20.8 - 40.2)			14.2 (8.5 - 20.9)		
L1	24.5 (19 - 34.4)	0.71 (0.4 - 1.27)	0.25	11 (4.9 - 14.5)	1.64 (0.98-2.76)	0.062
blood vessel invasion						
V0	26 (21.4 - 32.1)			12.8 (10 - 15.5)		
V1	34.4 (11.6 - 46.8)	0.79 (0.42 - 1.49)	0.473	5.6 (1.8 - 14.7)	1.12 (0.6-2.08)	0.729
resection margin						
R0	29.3 (24.2 - 37.9)			12.9 (10 - 16.4)		
R1	16.6 (11.5 - 20.8)	2.34 (1.36 - 4.04)	0.002	10.7 (4.1 - 13.3)	1.52 (0.92-2.5)	0.1
adjuvant therapy						
no	18.7 (11.5 23.7)			3.6 (1.6 - 5.2)		
yes	37.9 (28.5 - 49.4)	0.33 (0.21 - 0.53)	<0.001	15.5 (13.3 - 20.4)	0.25 (0.16-0.39)	<0.001
aRHA – aberrant right hepatic artery, CI – confidence interval, DFS – disease free survival, HR - hazard ratio, NA – not available, OS – overall survival						

### Hepatic recurrence

Complete follow-up of at least 24 months after resection was present in 91 out of 133 patients (68.4%). There were 13 out 91 (14.3%) patients with an aRHA. The estimated median follow-up in this subgroup was 63.3 (95 %CI 39.2-71.8) months. The overall recurrence rate among those 91 patients was 91.2%. The hepatic recurrence rate was 25.3%, local 30.8%, pulmonary 12.1%, other site 6.6%, and multiple sites 16.4%. The hepatic recurrence rate in the group of aRHA patients was 30.8% versus 24.4% in the group of conventional anatomy (odds ratio 1.38, 95% CI 0.34 - 4.78, p = 0.623). In the multivariable analysis, only lymphatic vessel invasion was an independent prognostic factor for hepatic recurrence [Table 4].

**Table 4:** Multivariable logistic regression of hepatic recurrence (N=91)

Parameter	N (% of total)	Hepatic recurrence (%)	OR (95% CI)	p - value
aRHA				
not present	78 (85.7)	19 (24.4)		
present	13 (14.3)	4 (30.8)	1.36 (0.3 - 5.38)	0.669
pT				
T1	17 (18.7)	5 (29.4)		
T2	55 (60.4)	15 (27.3)	0.67 (0.15 - 3.1)	0.601
T3/T4	19 (20.9)	3 (15.8)	0.32 (0.04 - 2.3)	0.269
pN				
N0	30 (33)	7 (23.3)		
N1	32 (35.2)	7 (21.9)	0.34 (0.06 - 1.61)	0.19
N2	29 (31.9)	9 (31)	0.42 (0.07 - 2.31)	0.333
grade				
G1/G2	62 (68.1)	11 (17.7)		
G3	29 (31.9)	12 (41.4)	2.89 (0.95 - 9.09)	0.062
lymphatic vessel invasion				
L0	36 (39.6)	4 (11.1)		
L1	55 (60.4)	19 (34.5)	7.33 (1.6 - 45.38)	0.017
blood vessel invasion				
V0	78 (85.7)	19 (24.4)		
V1	13 (14.3)	4 (30.8)	0.54 (0.1 - 2.62)	0.452
resection margin				
R0	72 (79.1)	18 (25)		
R1	19 (20.9)	5 (26.3)	1.06 (0.22 - 4.69)	0.935
adjuvant therapy				
no	23 (25.3)	7 (30.4)		
yes	68 (74.7)	16 (23.5)	0.67 (0.2 - 2.39)	0.524
aRHA – aberrant right hepatic artery, CI – confidence interval, OR - odds ratio				

## Discussion

In this retrospective, single-center study, we analyzed the impact of aRHA on the oncologic outcome after resection for PDAC of the pancreatic head. We showed no significant difference in OS, DFS, and hepatic recurrence between patients with aRHA and typical predominant liver vascular anatomy.

In a comprehensive review of the clinical significance of variant hepatic artery in pancreatic resection, Xu et al. showed that most studies on the subject report an incidence of aRHA between 10 and 24%.[12] Reviewing thoroughly the operation reports and the tomography imaging, we identified the presence of an aRHA in 14.5% of the patients, which falls in the range Xu et al. reported. We demonstrated no association of aRHA with other common clinical and histopathological parameters, especially, there was no difference in the rate of major postoperative complications Clavien-Dindo ≥3.[13]

Similarly, there was no difference in long-term outcome. Most other studies on the subject also reported no difference in oncologic outcome.[5, 6, 8, 14–18] Turrini et al showed poorer survival outcome for patients with involvement of the aRHA.[19] Of note, in our study none of the patients had tumor infiltration of the aRHA and there was only one case with the tumor abutting the aRHA, so we could not further explore this issue.

In a recent study, Mangieri et al. focused on the hepatic recurrence rate, demonstrating significantly higher hepatic recurrence rate of 61.8% in patients with an aRHA, compared to 25.3% in patient with conventional vascular anatomy.[9] In our study, we detected a hepatic recurrence rate of 30.8% in patients with aRHA versus 24.4% in patients with conventional anatomy, the difference being not statistically significant. In a meta-analysis of over 17 000 patients, the overall initial hepatic recurrence rate after resection for PDAC has been estimated to be around 26.5 %.[20] Again, this implies the plausibility of our results. In the cohort of Mangieri et al. not only PDAC but also ampullary and distal bile duct carcinoma were included, which might account for some of the difference in the results. Yet, in the case of PDAC, we could not confirm the association of aRHA with hepatic recurrence.

The major limitation of our study is its retrospective design. Since preoperative imaging and operation reports were available for all patients, we could objectively identify the presence of an aRHA retrospectively. Incomplete data on recurrence, which is a common problem in survival analysis, also poses a potential bias. In accordance to similar studies, we excluded patients with incomplete follow-up of less than 24 months.[21] In this cohort we demonstrated overall recurrence rate of 91% at median follow-up of 63.3 months. Similarly, Strobel et al. reported that less than 10 % of patients after upfront surgery for PDAC are recurrence-free after 5 years.[22] Further, circumferential margin status was not available for all patients, so we could not include this important prognostic parameter in the analysis.

The results of this study show no higher recurrence risks for patients with aRHA, so we do not advocate neoadjuvant therapy in patients with aRHA and otherwise resectable tumor. Systematic evaluation of the preoperative imaging should always be performed and in case of tumor contact to the aRHA, neoadjuvant therapy should be considered. Due to the small number of patients, we could not analyze the impact of neoadjuvant therapy in this study. Further research on the differential influence of neoadjuvant therapy in patients with aRHA and PDAC of the pancreatic head is warranted. Though in none of the patients in this cohort an arterial resection of the aRHA for oncological reason was performed, we consider it challenging and with high risk of complications, so we would not recommend it. Further studies on the influence of aRHA on the oncological outcome of other periampullary carcinomas such as distal bile duct carcinoma and ampullary carcinoma will be of interest.

## Conclusion

There is a relevant proportion of patients undergoing pancreatic resection that have aberrant vascular anatomy of the liver. The presence of an aRHA does not seem to influence the long-term outcome after resection for PDAC of the pancreatic head.

## Data Availability

The data that support the findings of this study are not openly available due to reasons of sensitivity and are available from the corresponding author upon reasonable request and with permission from the University Hospital, Goethe University Frankfurt.
